# A simplified lung ultrasound for the diagnosis of interstitial lung disease in connective tissue disease: a meta-analysis

**DOI:** 10.1186/s13075-019-1888-9

**Published:** 2019-04-11

**Authors:** Hai Qin Xie, Wei Wei Zhang, De Sheng Sun, Xiang Mei Chen, Shu Fang Yuan, Zheng Hua Gong, Li Liu

**Affiliations:** Department of Ultrasound, Peking University Shenzhen Hospital, Lianhua Road 1120, FuTian District, Shenzhen, 518036 Guangdong China

**Keywords:** Lung ultrasound, Interstitial lung disease, Connective tissue disease, Meta-analysis

## Abstract

**Background:**

Interstitial lung disease (ILD) is a common complication of connective tissue disease (CTD) and a leading cause of morbidity and mortality. There are various lung ultrasound (LUS) scoring systems with different lung intercostal spaces (LIS). The purpose of this meta-analysis was to find a simplified LUS method for the assessment of CTD-ILD.

**Methods:**

We systematically retrieved lung ultrasound diagnostic studies on CTD-ILD in PubMed, Embase, and Web of Science databases. Summary diagnostic accuracy, including sensitivity, specificity, and area under the curve (AUC), was analyzed. Subgroup analysis was conducted according to different LIS and diseases.

**Results:**

The 11 studies included in this meta-analysis comprised a total of 487 patients with CTD. The pooled sensitivity and specificity of the LUS were 0.859 (95% confidence interval (CI) 0.812–0.898) and 0.839 (95% CI 0.782–0.886), respectively, illustrating its great value for CTD-ILD diagnosis. In addition, there were six methods to evaluate LIS, including 72, 65, 50, 14, 10, and all LIS. The pooled sensitivity and specificity of 14 LIS were 0.982 (95% CI 0.904–1.000) and 0.875 (95% CI 0.710–0.965), respectively. The pooled positive likelihood ratio (PLR), negative likelihood ratio (NLR), and diagnostic odd ratio (DOR) of 14 LIS were 7.297 (95% CI 6.050–17.45), 0.029 (95% CI 0.006–0.147), and 292.30 (95% CI 35.53–2403.8), respectively. Moreover, the AUC for systemic sclerosis (SSc) and rheumatoid arthritis (RA) was 0.929 and 0.981, respectively; the DOR for SSc and RA was 42.93 (95% CI 17.75–103.79) and 80.24 (95% CI 8.107–796.09), respectively.

**Conclusions:**

We found a modified and simplified method of LUS, by scanning 14 LIS in a short time, which had a very high sensitivity and specificity.

## Introduction

Interstitial lung disease (ILD) is a common complication of connective tissue disease (CTD) and a leading cause of morbidity and mortality [1]. Thus, early diagnosis and treatment may improve the prognosis of patients with ILD [2].

High-resolution computed tomography (HRCT) is the gold standard for ILD diagnosis [3–5]. It can detect the location and type of lesions through its high resolution. Unfortunately, it is hampered by high cost and potential risks associated with radiation exposure, especially for pregnant women. Accordingly, finding a low-cost, non-invasive, and non-ionizing diagnostic method is necessary for ILD. Lung ultrasound (LUS) has all of these advantages and is an accessible bedside procedure. As a result, it is easily accepted by patients. Over the last 20 years, LUS has mainly been applied in CTD-ILD diagnosis, where it has shown high sensitivity and specificity. The assessment of ILD by LUS is determined by the number of B-lines, which appear as a comet tail signal and originate from the pleural line without fading to the edge of the screen [6].

The total number of B-lines was found to correlate well with the HRCT score [7]. To assess the number of B-lines, previous studies used various scoring systems by designing different intercostal spaces (LIS), such as 72 LIS, 50 LIS, and 14 LIS [7–10]. In fact, 70 and 50 LIS were time-consuming and hard to perform daily. Up to now, there have been few data about which LIS should be better studied for calculating the number of B-lines by LUS. Accordingly, a meta-analysis is needed to find a simplified LUS method for CTD-ILD diagnosis.

## Methods

### Search strategy and selection studies

The PubMed, Embase, and Web of Science databases were searched up to October 31, 2018. Two investigators independently searched the databases and screened the articles. Disagreements were resolved by a third investigator. All studies found were in English. We used various combinations of Medical Subject Heading (MSH) terms, including ultrasound, sonography, lung, interstitial, pulmonary fibrosis, and connective tissue disease. The search string also included B-line and high-resolution computed tomography. Selected studies were about the LUS diagnostic value according to the B-lines in patients with CTD-ILD, compared to HRCT. All the references mentioned in the selected studies were reviewed to avoid omitting studies not indexed by the electronic databases. Articles with overlapping data or insufficient data, conference abstracts, reviews, and meta-analyses were excluded.

### Data extraction and quality assessment

Data from the included studies were extracted independently by two researchers and consisted of the characteristic features, such as the author, publication year, country, patients’ sex and mean age, number of LIS, cutoff values of the B-lines, probe frequency, probe type, mean disease duration, number of LUS operators, and kappa value. True positive, false positive, true negative, and false negative were obtained from the selected studies. LUS diagnosis of CTD-ILD was scored by the number of B-lines, using HRCT as the golden standard. The quality of each article was evaluated by means of Quality Assessment of Diagnostic Accuracy Studies (QUADAS) [11].

### Statistical analysis

The heterogeneity was evaluated using the *I*^*2*^ statistic. When the *I*^*2*^ of heterogeneity was greater than 50, we used the random effect model. Otherwise, the fixed effect model [12] was used. Summary sensitivity, specificity, positive likelihood ratio (PLR), negative likelihood ratio (NLR), and diagnostic odds ratio (DOR) were measured. The 95% confidence interval (CI) was calculated for individual and pooled data. In addition, we analyzed the summary receiver-operating characteristic curves (SROC), area under the curve (AUC), and *Q* index for all studies. The subgroup analysis was performed according to the number of LIS and different diseases. The software used was Meta-DiSc (version 1.4, Ramon y Cajal Hospital, Madrid, Spain) [13].

## Results

### Literature inclusion and data extraction

**Fig. 1:**
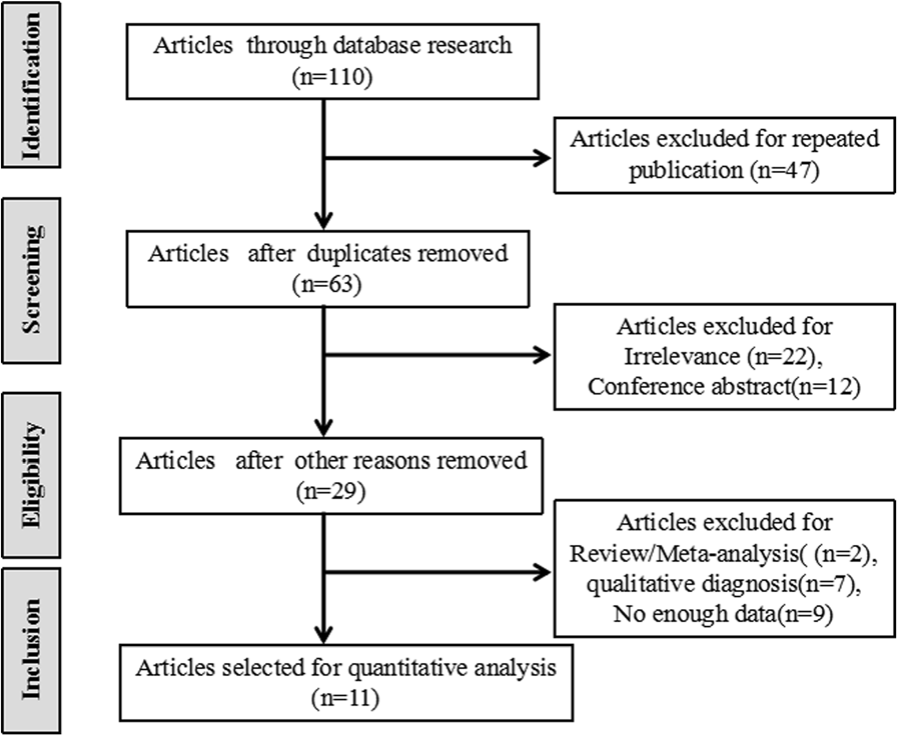
A flow diagram showing article selection

We retrieved 110 articles in the primary search. After reading the titles and abstracts, 29 articles were selected for reviewing the full text. Two studies were omitted because they were review or meta-analysis. Seven studies were omitted due to the qualitative diagnosis. Three studies were omitted because they mainly compared two LUS methods. The results indicated a highly significant correlation between two methods without the data about the diagnostic accuracy [14–16]. Three studies were deleted because they described a good correlation between B-line score of LUS and the Warrick score on HRCT, but no sensitivity and specificity data were provided [17–19]. Three studies were excluded because they mainly focused on the diagnosis of ILD by LUS with the sign of pleural irregularity, not with the number of B-lines [20–22]. Ultimately, 11 articles were included, comprising a total of 487 patients (Fig. 1). We extracted the data from the 11 articles (Tables 1 and 2) and measured the LUS diagnostic value in CTD-ILD. One study analyzed three diseases, including systemic lupus erythematosus (SLE), rheumatoid arthritis (RA), and systemic sclerosis (SSc), and separately calculated the diagnostic accuracy of each [23]. Ten of the 11 studies acquired the scores of QUADAS more than 10 (Table 3).

**Table 1:** Characteristics of individual articles selected

First author [Ref]	Year	Country	Disease	LIS	Numbers		Cutoff value (B-line)	Probe frequency (MHz)	Probe type	True positive	False positive	False negative	True negative	Study quality
					ILD (+)	ILD (−)								
Delle [32]	2010	Italy	SSc	65	16	11	5	2.5–3.5; 6–12	2.5–3.5 (cardiac sector probe); 6–12 (linear probe)	12	2	4	9	7
Tardella [10]	2012	Italy	CTD	50	26	8	10	2–7	Broadband convex multi-frequency transducer	20	0	6	8	13
Barskova [24]	2013	Italy	SSc	72	36	22	5	2.5–3.5	Cardiac sector transducer	36	10	0	12	13
Mohammadi [33]	2014	USA	SSc	10	53	17	5	7–10	Broadband linear multi-frequency transducer	39	2	14	15	11
Moazedi-Fuerst [34]	2014	Austria	RA	All	17	47	–	3.5	Convex transducer	16	2	1	45	11
Cogliati [7]	2014	Italy	RA	72	13	26	10	5–2	Convex probe	12	13	1	13	13
Moazedi-Fuerst(a) [23]	2015	Austria	RA	All	7	18	–	3.5	Convex transducer	7	1	0	17	10
Moazedi-Fuerst(b) [23]	2015	Austria	SSc	All	9	5	–	3.5	Convex transducer	9	2	0	3	10
Moazedi-Fuerst(c) [23]	2015	Austria	SLE	All	4	2	–	3.5	Convex transducer	4	0	0	2	10
Vizioli [35]	2016	Italy	ILD	All	25	24	5	5–8; 2–5	5–8 (linear probe); 2–5 (convex probe)	23	7	2	17	14
Cakir [29]	2016	Turkey	SSc	14	29	19	5	5–10	Linear probe	29	3	0	16	11
Vasco [8]	2017	Spain	SS	All	4	9	3	2–5.5; 3.3–10	2–5.5 (60-mm-wide-band convex array probe); 3.3–10 (linear array)	4	1	0	8	14
Tardella [9]	2018	Italy	SSc	14	27	13	10	4–13	Broadband linear transducer	26	1	1	12	14

a, b, and c represent one article including three different diseases

*ILD* interstitial lung disease, *CTD* connective tissue disease, *SSc* systemic sclerosis, *RA* rheumatoid arthritis, *SLE* systemic lupus erythematosus, *SS* Sjögren’s syndrome, *LIS* intercostal spaces, − not available

**Table 2:** Demographic characteristics of study populations

First author [Ref]	Numbers		Mean age (range) years ± standard deviation	Mean disease duration (months, years)	Spend time (min)	Number of LUS operator	Reproducibility between operator
	Female	Male					
Delle [32]	22	3	53 ± 10.5	–	–	2	Intra-class correlation = 0.681
Tardella [10]	30	4	57 ± 13	85.58 ± 84.37 months	23 ± 4.5	2	Kappa = 0.846–0.980
Barskova [24]	54	4	51 ± 14	–	< 10	2	Intra-observer variability 5.1%, inter-observer variability 7.4%
Mohammadi [33]	62	8	50.29 ± 9.7	88 ± 83.1 months	–	1	Kappa = 0.838
Moazedi-Fuerst [34]	54	10	59 ± 12	9.4 (2–21) years	–	2	Kappa = 0.92
Cogliati [7]	29	10	64.87	11.21 years	–	2	Kappa = 0.78
Moazedi-Fuerst [23]	38	7	54 (28–74)	8 (1–35) years	–	2	–
Vizioli [35]	–	–	65 ± 13		8 ± 1 (5–14)		–
Cakir [29]	46	2	50.8 ± 11.9	4.6 ± 3.8 years	–	2	Inter-observer reliability *r* = 0.96
Vasco [8]	13	0	63.62 (39–88)	–	–	2	Intra-rater reliability *k* = 1
Tardella [9]	34	6	56.4 ± 13.42	78 ± 81.52 months	8.7 ± 1.3	2	–

*LUS* lung ultrasound

**Table 3:** Study quality using the QUADAS tool

First author [Ref]	1	2	3	4	5	6	7	8	9	10	11	12	13	14	Sum
Delle [32]	Y	U	Y	U	Y	Y	Y	N	N	Y	U	Y	U	U	7
Tardella [10]	Y	Y	Y	Y	Y	Y	Y	Y	Y	Y	Y	Y	U	Y	13
Barskova [24]	Y	Y	Y	Y	Y	Y	Y	Y	Y	Y	U	Y	Y	Y	13
Mohammadi [33]	Y	U	Y	U	Y	Y	Y	Y	Y	Y	Y	Y	U	Y	11
Moazedi-Fuerst [34]	Y	Y	Y	U	Y	Y	Y	Y	N	Y	U	Y	Y	Y	11
Cogliati [7]	Y	Y	Y	U	Y	Y	Y	Y	Y	Y	Y	Y	Y	Y	13
Moazedi-Fuerst [23]	Y	N	Y	U	Y	Y	Y	Y	N	Y	U	Y	Y	Y	10
Vizioli [35]	Y	Y	Y	Y	Y	Y	Y	Y	Y	Y	Y	Y	Y	Y	14
Cakir [29]	Y	Y	Y	Y	Y	Y	Y	Y	Y	U	U	Y	N	Y	11
Vasco [8]	Y	Y	Y	Y	Y	Y	Y	Y	Y	Y	Y	Y	Y	Y	14
Tardella [9]	Y	Y	Y	Y	Y	Y	Y	Y	Y	Y	Y	Y	Y	Y	14

*QUADAS* Quality Assessment of Diagnostic Accuracy Studies, *Y* yes, *N* no, *U* unclear

### Diagnostic accuracy of LUS in different LIS

**Fig. 2:**
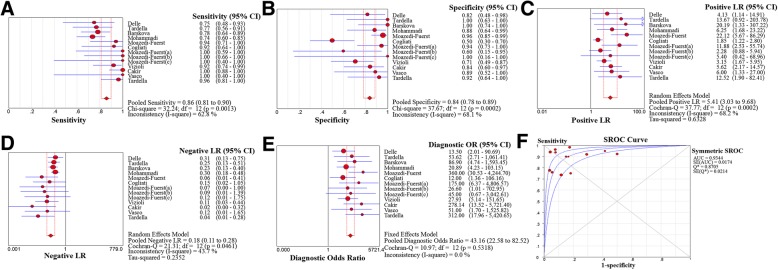
Forest plots of lung ultrasound for interstitial lung disease diagnosis in patients with connective tissue disease. Sensitivity (**a**), specificity (**b**), positive likelihood ratio (**c**), negative likelihood ratio (**d**), diagnostic odds ratio (**e**), and summary receiver operative curves (**f**)

**Fig. 3:**
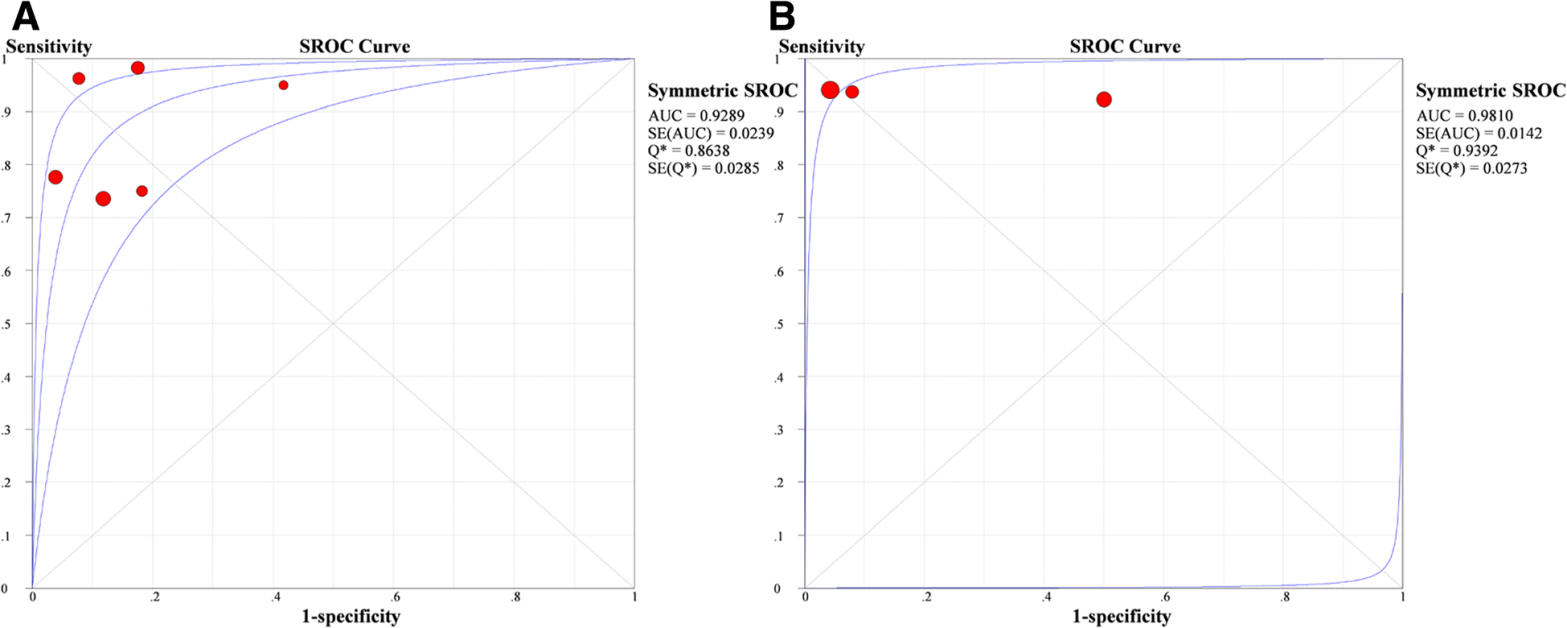
Summary receiver-operating characteristic curves for lung ultrasound for interstitial lung disease diagnosis of systemic sclerosis (**a**) and rheumatoid arthritis (**b**). Red dots represent individual articles included in our meta-analysis. SE (AUC), standard error of the area under the curve; Q*, an index defined by the point on the SROC curve where the sensitivity and specificity are equal; SE (Q*), Q* index standard error

A total of 11 articles were pooled together to calculate the summary diagnostic accuracy of LUS in patients with CTD-ILD (Table 4 and Fig. 2). The sum of patients with ILD was 266 and 221 without ILD. The pooled sensitivity and specificity of LUS were 0.859 (95% CI 0.812–0.898) and 0.839 (95% CI 0.782–0.886), respectively. In general, the pooled PLR, NLR, and DOR were 5.412 (95% CI 3.026–9.680), 0.176 (95% CI 0.111–0.279), and 43.16 (95% CI 22.58–82.52), respectively. The AUC of LUS was 0.934, and the Q^*^ index was 0.871 (Fig. 3), illustrating its great value for CTD-ILD diagnosis.

**Table 4:** Diagnostic accuracy of LUS in different LIS

Different LIS	Study no.	Number		Sensitivity (95% CI)	Heterogeneity		Specificity (95% CI)	Heterogeneity		PLR (95% CI)	NLR (95% CI)	DOR (95% CI)	AUC (SE)	Q* (SE)
		ILD (+)	ILD (−)		*I* ^2^	*p* value		*I* ^2^	*p* value					
Total	11	266	221	0.859 (0.812–0.898)	62.8	0.001	0.839 (0.782–0.886)	68.1	0	5.412 (3.026–9.680)	0.176 (0.111–0.279)	43.16 (22.58–82.52)	0.934 (0.017)	0.871 (0.021)
All	4	68	105	0.955 (0.873–0.991)	0	0.721	0.876 (0.798–0.932)	59.6	0	5.476 (2.597–11.55)	0.086 (0.035–0.211)	59.76 (21.05–1525.8)	0.975 (0.016)	0.927 (0.027)
72	2	49	48	0.814 (0.691–0.903)	35.0	0.215	0.358 (0.486–0.804)	92.2	0	3.367 (1.810–6.621)	0.208 (0.110–0.396)	25.57 (5.183–146.7)	–	–
65	1	16	11	0.75 (0.48–0.93)	–	–	0.82 (0.48–0.98)	–	–	4.13 (1.14–14.91)	0.31 (0.13–0.75)	13.50 (2.01–90.69)	–	–
50	1	26	8	1.000 (0.398–1.000)	–	–	1.000 (0.158–1.000)	–	–	5.400 (0.423–68.96)	0.120 (0.008–1.746)	45.00 (0.666–3042.6)	–	–
14	2	56	32	0.982 (0.904–1.000)	32.4	0.224	0.875 (0.710–0.965)	0	0.485	7.297 (3.050–17.45)	0.029 (0.006–0.147)	292.30 (35.53–2403.8)	–	–
10	1	53	17	0.736 (0.597–0.847)	–	–	0.882 (0.636–0.985)	–	–	6.255 (1.685–23.223)	0.299 (0.185–0.485)	20.893 (4.232–103.15)	–	–

The number 1 means 100% in sensitivity and specificity

*LIS* intercostal spaces, *ILD* interstitial lung disease, *PLR* positive likelihood ratio, *NLR* negative likelihood ratio, *DOR* diagnostic OR, *AUC* area under the curve, *CI* confidence interval, *SE* standard error, − not available

There were six different methods of LIS in the 11 articles, including 72, 65, 50, 14, 10, and all LIS (Table 4). The pooled sensitivity, specificity, PLR, NLR, DOR, and AUC of all LIS were 0.955 (95% CI 0.873–0.991), 0.876 (95% CI 0.798–0.932), 5.476 (95% CI 2.597–11.55), 0.086 (95% CI 0.035–0.211), 59.76 (95% CI 21.05–1525.8), and 0.975, respectively. The pooled sensitivity, specificity, PLR, NLR, and DOR of 14 LIS were 0.982 (95% CI 0.904–1.000), 0.875 (95% CI 0.710–0.965), 7.297 (95% CI 3.050–17.45), 0.029 (95% CI 0.006–0.147), and 292.30 (95% CI 35.53–2403.8), respectively. All LIS and 14 LIS both had high sensitivity and specificity. In the search for a simplified and less time-consuming method, 14 LIS was found to be the best choice for LUS assessment in patients with CTD-ILD.

### Diagnostic value of LUS in different diseases

There were six articles on SSc and three articles on RA (Table 5). The pooled sensitivity, specificity, PLR, NLR, and DOR of SSc were 0.839 (95% CI 0.777–0.889), 0.870 (95% CI 0.774–0.936), 6.203 (95% CI 3.565–10.800), 0.191 (95% CI 0.098–0.373), and 42.93 (95% CI 17.75–103.79), respectively. The pooled sensitivity, specificity, PLR, NLR, and DOR of RA were 0.946 (95% CI 0.818–0.993), 0.824 (95% CI 0.730–0.896), 7.398 (95% CI 0.768–74.220), 0.082 (95% CI 0.024–0.275), and 80.24 (95% CI 8.107–796.09), respectively. The AUC and the Q^*^ index of SSc were 0.923 and 0.864, respectively, while the AUC and Q^*^ index of RA were 0.981 and 0.939, respectively (Fig. 3), indicating that LUS is a very useful tool for SSc and RA diagnosis.

**Table 5:** Summary diagnostic accuracy of LUS in different diseases

Disease	Study no.	Numbers		Sensitivity (95% CI)	Heterogeneity		Specificity (95% CI)	Heterogeneity		PLR (95% CI)	NLR (95% CI)	DOR (95% CI)	AUC (SE)	Q* (SE)
		ILD (+)	ILD (−)		*I* ^2^	*p* value		*I* ^2^	*p* value					
SSc	6	170	87	0.839 (0.777–0.889)	78.3	0	0.870 (0.774–0.936)	21.4	0.272	6.203 (3.565–10.800)	0.191 (0.098–0.373)	42.93 (17.75–103.79)	0.929 (0.024)	0.864 (0.029)
RA	3	37	91	0.946 (0.818–0.993)	0	0.636	0.824 (0.730–0.896)	91.8	0	7.398 (0.768–74.220)	0.082 (0.024–0.275)	80.24 (8.107–796.09)	0.9810 (0.014)	0.939 (0.027)

*CTD* connective tissue disease, *ILD* interstitial lung disease, *SSc* systemic sclerosis, *RA* rheumatoid arthritis, *PLR* positive likelihood ratio, *NLR* negative likelihood ratio, *DOR* diagnostic OR, *AUC* area under the curve, *CI* confidence interval, *SE* standard error, − not available

## Discussion

During the last decade, numerous studies on the role of LUS for ILD diagnosis in patients with CTD have been reported. Semi-quantitative data were measured by the sum of the number of B-lines, which were counted by the designed LIS [7, 9, 24]. However, there were extensive LUS scoring systems to assess the B-lines. Some studies referred to more LIS, such as all LIS, 72 LIS, and 50 LIS, which were time-consuming and difficult to extend [7, 10]. Besides, without a uniform criteria method, it was hard to spread. Currently, there is no evidence about which LIS should be observed. Therefore, it is necessary to find a simplified and uniform method with fewer LIS for improved LUS diagnostic performance.

With the development of ultrasound technology, more and more studies have focused on ultrasound in lung diseases, which include pneumonia [25], neonatal respiratory distress syndrome [26], and interstitial lung disease [27]. In our meta-analysis, we concentrated on LUS in the diagnosis of ILD in patients with CTD. In a total of 487 patients with CTD, nearly half had the complication of ILD. The overall AUC was 0.934, indicating a high level of diagnostic performance. Our finding was similar to that in a recent study [28]. However, there were some differences between the two meta-analysis studies. First, in this meta-analysis, the emphasis was on finding a simplified LUS method, to facilitate daily clinical management, using the same uniform diagnostic criteria. Second, our meta-analysis included more studies than that of Song et al. Our meta-analysis included 11 studies with a total of 487 patients. The study by Song et al. included 6 studies with a total of 272 patients. Besides, more studies from the last 3 years were included in our study. Third, our study included more different diseases in CTD, such as Sjögren’s syndrome.

This meta-analysis found that all LIS and 14 LIS both had high diagnostic value. However, all LIS was time-consuming and not available for clinical practice. Therefore, 14 LIS may be the better choice for LUS diagnosis. 14 LIS consisted of bilateral, anterior, and posterior locations. For the unilateral chest, they selected 4 LIS including the second LIS on the parasternal lines and the fourth LIS on midclavicular, the anterior axillary, and the midaxillary lines respectively. For the posterior chest, they selected the eighth LIS on three lines, namely the paravertebral, the subscapular, and the posterior axillary lines. Among the reasons for choosing these LIS were the demonstrated higher prevalence rate of B-lines in these fields and the ease of evaluation by LUS [14, 29]. To the best of our knowledge, there is no meta-analysis about which LIS should be evaluated in a modified LUS for diagnosis of CTD-ILD. We found that the overall diagnostic performance of 14 LIS was higher than that of 72 LIS. The possible reasons may be associated with different diagnostic criteria, disease duration, deviations among patients, etc.

We also performed subgroup analysis by diseases. Since ILD is common in patients with SSc [30] and RA [31], most of the studies were focused on these two diseases. In our study, the results were not significantly different between them, showing that LUS was of a great diagnostic value for both diseases. In comparison, the diagnostic efficiency of LUS in RA was slightly higher than SSc. The DOR and AUC of RA were 80.24(95% CI 8.107–796.09) and 0.981, respectively. The DOR and AUC of SSc were 42.93(95% CI 17.75–103.79) and 0.929, respectively. The difference was possibly related to the different observed LIS, probe types, probe frequency, disease duration, etc. The number of LIS observed in patients with RA was all and 72 LIS, but in patients with SSc was diverse, including all, 72, 65, 14, and 10 LIS. The number of studies in SSc was more than that in RA. Moreover, the number of patients with SSc was larger than those with RA. Above all, the overall accuracy was relatively lower in SSc, but it still demonstrated that LUS was a useful method for the diagnosis of ILD in patients with SSc.

There was heterogeneity in this study. For the diagnostic accuracy in different LIS, the *I*^2^ of all LIS and 72 LIS were 59.6 and 92.2 in specificity, respectively. The reasons may be connected with different diseases, territory, and basic characteristic information. For the summary diagnostic accuracy in all studies, there was also heterogeneity in sensitivity and specificity. It is possible that it was related to demographic characteristics, such as probe types, probe frequency, number of LIS, disease duration, and reproducibility between operators.

Our meta-analysis had some limitations. First, the search strategy was restricted to studies in the English language. So, certain studies were missing. Second, there was no uniform standard set of criteria for ILD diagnosis by LUS. The result of sensitivity and specificity showed considerable variation. The sensitivity of our study was from 73.6 to 100%, and specificity was from 50 to 100%. The pooled data produced a more reliable result and decreased the variation. The study quality was evaluated using the tool of QUADAS. The quality of some studies was low, which would have an impact on our results. Third, there was heterogeneity among the articles, but we performed subgroup analysis, to find the possible reasons. Fourth, the number of studies was small; thus, there was not enough data to prove the diagnostic accuracy.

## Conclusions

We found a modified and simplified method of LUS, by scanning 14 LIS in a short time, which had a very high sensitivity and specificity. This LUS method may be a good choice for the assessment of ILD in patients with CTD. However, our data had some limitations, and more evidence is needed.

## Abbreviations

AUC: Area under the curve
CI: Confidence interval
CTD: Connective tissue disease
DOR: Diagnostic OR
HRCT: High-resolution computed tomography
ILD: Interstitial lung disease
LIS: Intercostal spaces
LUS: Lung ultrasound
MSH: Medical Subject Headings
NLR: Negative likelihood ratio
PLR: Positive likelihood ratio
QUADAS: Quality Assessment of Diagnostic Accuracy Studies
RA: Rheumatoid arthritis
SE: Standard error
SLE: Systemic lupus erythematosus
SROC: Summary receiver-operating characteristic curves
SS: Sjögren’s syndrome
SSc: Systemic sclerosis
